# A Case of Verminous Disease

**Published:** 1853-05

**Authors:** J. Windle

**Affiliations:** Oskaloosa, Iowa


### Art. IV.—*A Case of Verminous Disease.* By J. Windle, M. D., of Oskaloosa, Iowa.

November, 1852, I was consulted about a lad, fourteen years old, the son of Mr. Durbin, of Lane county, Ky. His father gave me the following history of his case: In December, of the previous year, the boy began to show indisposition, and on the night of the 25th, after retiring to bed, apparently not more out of health than usual, he was seized with difficult, loud breathing, and was discovered to be nearly pulseless, flaccid, and unconscious. On falling asleep, after being aroused, the same symptoms recurred several times during the night. Vermifuges were administered, by the advice of a physician, but without any good effect.

These attacks continued to recur, at irregular intervals, invariably at night, two or three hours after retiring; sometimes every night, then every third or sixth night.

In March, 1852, when I saw him, he was much emaciated, abdomen slightly tumid, upper lip a little swollen, tongue coated with a white fur, the papillæ prominent and red. Calomel grs. xv., and gamboge grs. iii., were prescribed two nights in succession. The action of the purgative was brisk, and several hundred small worms, of a peculiar form and appearance, were brought away. They are not of any species usually found in the intestinal canal of the human subject, but of a dark brown, almost black color, round in form, tapering an inch or less in length, and two lines in thickness. The relief from the expulsion of the worms was complete, and the lad was soon restored to perfect health.

The facts of this case illustrate well the influence of intestinal irritation upon the nervous centres, of which the practice of physic daily furnishes so many examples.

March, 1853.

